# Linear dichroism of visible-region chromophores using M13 bacteriophage as an alignment scaffold†

**DOI:** 10.1039/c8ra05475d

**Published:** 2018-08-20

**Authors:** Matthew Tridgett, Charles Moore-Kelly, Jean-Louis H. A. Duprey, Lorea Orueta Iturbe, Chi W. Tsang, Haydn A. Little, Sandeep K. Sandhu, Matthew R. Hicks, Timothy R. Dafforn, Alison Rodger

**Affiliations:** School of Biosciences, University of Birmingham Edgbaston Birmingham West Midlands B15 2TT UK mxt133@bham.ac.uk; School of Chemistry, University of Birmingham Edgbaston Birmingham West Midlands B15 2TT UK; Department of Molecular Sciences, Macquarie University Sydney NSW 2109 Australia

## Abstract

It is a challenge within the field of biomimetics to recreate the properties of light-harvesting antennae found in plants and photosynthetic bacteria. Attempts to recreate these biological structures typically rely on the alignment of fluorescent moieties *via* attachment to an inert linear scaffold, *e.g.* DNA, RNA or amyloid fibrils, to enable Förster resonance energy transfer (FRET) between attached chromophores. While there has been some success in this approach, refinement of the alignment of the chromophores is often limited, which may limit the efficiency of energy transfer achieved. Here we demonstrate how linear dichroism spectroscopy may be used to ascertain the overall alignment of chromophores bound to the M13 bacteriophage, a model linear scaffold, and demonstrate how this may be used to distinguish between lack of FRET efficiency due to chromophore separation, and chromophore misalignment. This approach will allow the refinement of artificial light-harvesting antennae in a directed fashion.

## Abstract 2

Here we characterise four dyes and assess the complementarity of linear dichroism and FRET in biomimetic light-harvesting antennae optimisation.

## Graphical abstract

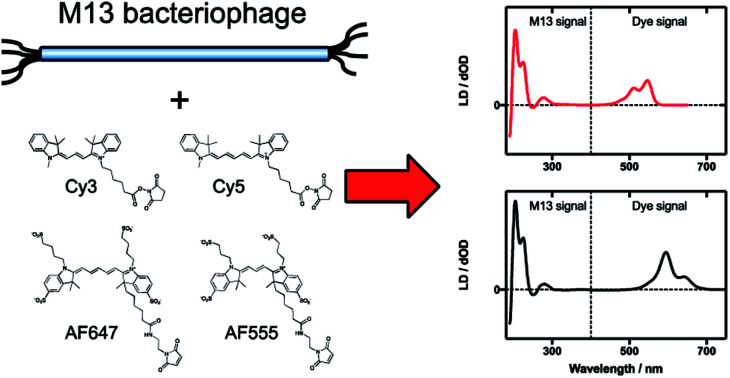

## Introduction

Natural light-harvesting complexes (LHCs) harvest energy from the sun leading to its transformation into a form that can be stored and used. They function as a result of highly ordered arrangements of biological chromophores called accessory pigments, which funnel light energy towards a reaction centre, which captures the energy in chemical form.^1^ Pigment dipoles within photosynthetic organisms, including purple bacteria and green plants, are anisotropically organised within LHCs, which are approximately cylindrical transmembrane pigment–protein complexes.^2–5^ The high degree of anisotropy of pigments within LHCs enables efficient energy transfer between chromophores and thus highly efficient light harvesting. LHC pigment organisation has been investigated using a variety of techniques, including X-ray crystallography,^2^ circular dichroism,^6,7^ and linear dichroism.^8,9^ In turn, the supramolecular organisation of LHCs has been investigated using, for example, electron microscopy and atomic force microscopy.^10,11^

This concept is extremely attractive and researchers have often tried to mimic ordered arrangements of chromophores to achieve the same light harvesting effect *in vitro*. Approaches include the use of self-assembling nanoparticles to organise light-harvesting chromophores,^12^ pigment micellisation,^13^ self-assembly of chromophore-functionalised cellulose nanorods,^14^ macromolecular porphyrin self-assembly,^15^ self-assembly of light-harvesting dendrimers,^16^ and the construction of two-dimensional covalent organic frameworks of pigments.^17^ What is common to all these approaches is the attempt to organise pigments anisotropically, thus mimicking natural LHCs.

An approach often taken to mimic natural LHCs involves the use of a high aspect ratio scaffold onto which a large number of chromophores are bound to allow unidirectional transfer of energy *via* resonance energy transfer, mimicking the light-capturing antennae that surround reaction centres in plants and photosynthetic bacteria. High aspect ratio scaffolds used for the ordered arrangement of chromophores include: DNA;^18^ the coat proteins of the filamentous plant virus Tobacco Mosaic Virus (TMV);^19^ M13 bacteriophage;^20^ peptide nanotubes;^21^ and amyloid-like protein fibrils.^22^

While these techniques yielded functioning light-capturing antennae, there was often limited optimisation of the alignment of chromophores, or of the antennae, and surprisingly, a number of assumptions were made regarding the orientation of dyes associated with linear scaffolds: Dutta *et al.*^18^ relied on fluorescence anisotropy to demonstrate that dyes bound to DNA were not freely rotating and thus assumed that they were bound with strongly aligned transition dipoles. While it was mentioned that more detailed analysis would be required to determine the actual geometries of the dyes, no such analysis was provided. In contrast, Miller *et al.*^19^ assumed random orientation of chromophores on TMV coat proteins. They could have had greater insight and potential to optimise energy transfer by investigating the alignment of the dyes relative to the scaffold. Nam *et al.*^20^ did not optimise orientation of dyes bound to M13 bacteriophage; they relied on the flexibility of the N-terminus of the coat protein to enable energy transfer between dyes. This could have been optimised if information were available to determine the orientation of the dyes on the bacteriophage. Matsuie and MacCuspie used infrared spectroscopy to infer the orientation of porphyrins bound to the surface of a peptide nanotube.^21^ While they were able to suggest an orientation, the model they proposed was only one of a number of possibilities. Finally, Channon *et al.*^22^ relied on the assumption that the binding of fluorophores to a rod-like scaffold provides rigid orientation, a claim that could have been supported experimentally were information about the alignment of the dyes on the nanotubes available. It is clear from these examples that, were information regarding the alignment of the dyes relative to the scaffold available, rational optimisations of the dye scaffolds could be made to enable improved alignment of dye transition dipole moments, a requirement for optimal FRET efficiency and thus optimal light harvesting.

In this work we have used M13 bacteriophage as a well-defined biological scaffold to align light harvesting chromophores. We have used linear dichroism (LD) spectroscopy, a well-established technique,^23–30^ as a method to measure the alignment of visible-region chromophores bound to the biological scaffold. The method demonstrated here rapidly provides orientation information that can be used in directed optimisations of the dye arrays.

In order to show the versatility of our approach we illustrate how it can be used to assess the alignment of Cyanine3, Cyanine5 and Alexa Fluors 555 and 647, assembled on M13 bacteriophage as a scaffold. We used the stretched-film LD technique developed by Razmkhah *et al.* to determine the transition dipole polarisations of the dyes, which are needed for the analysis.^31^

## Experimental

### Materials

Cyanine3 NHS ester and Cyanine5 NHS ester were obtained from Lumiprobe GmbH (Germany). Alexa Fluors 555 C_2_-maleimide and 647 C_2_-maleimide were obtained from Invitrogen (USA). The film stretcher was built at the University of Warwick, UK. The film was cut from KitKare Ltd (UK) Clear Polythene Plastic Bags (120 gauge, 30 μm).

### Mass spectrometry

Alexa Fluor 555 C_2_-maleimide was dissolved in 50 : 50 water : acetonitrile with 0.5% triethylamine. Negative electrospray ionisation time of flight mass spectrometry was performed at the Centre for Chemical and Material Analysis at the University of Birmingham.

### Stretched-film linear dichroism measurements

3 × 5 cm^2^ polyethylene (PE) films were cut from KitKare Ltd Clear Polythene Plastic Bags (120 gauge, 30 μm). The films were then treated in a Harrick Plasma Cleaner PDC-32G-2 set to RF level HI for 4 minutes to produce oxidised PE (PE^ox^). The PE^ox^ films were then clamped into a film stretcher built at the University of Warwick, with the clamps set at a separation of 2.5 cm and the film oriented with the manufacturing stretch direction aligned with the film stretcher stretching direction. In some experiments the film was stretched before adding the sample thus enabling the baseline to be collected on the same film. In others (where stretch was the variable being considered) the sample was added to the unstretched film and then stretched and the baseline was a different film stretched to the same extent. The dye was then dried under vacuum for a further 15 minutes. The standard stretch amount was 1.8× its original length, 4.5 cm, unless the effect of stretching was being studied. All samples were measured using a Jasco J-1500 Circular Dichroism Spectrometer (parameters for each dye are provided in the ESI†).

### Production of M13 bacteriophage

M13 bacteriophage stocks were grown using One Shot™ TOP10F′ *Escherichia coli* (Thermo Fisher Scientific) as the host, and purified as previously published.^32^

### Bacteriophage-dye conjugation

Cyanine3 NHS ester (Cy3) or Cyanine5 NHS ester (Cy5) was dissolved to 10 mg mL^−1^ in dimethyl sulfoxide (DMSO). To 980 μL of M13 bacteriophage at 2.5 mg mL^−1^ in 50 mM potassium phosphate buffer, pH 8.0 in a 1.5 mL microcentrifuge tube, 20 μL of a dye solution was added. Following overnight incubation in the dark at 20 °C with slow mixing, unbound dye was removed from the mixture using a PD midiTrap G-25 column (GE Healthcare), eluting into 1.5 mL of 50 mM potassium phosphate buffer, pH 8.0. When the bacteriophage was labelled with both Cy3 and Cy5, the same protocol was followed, simultaneously adding half the concentrations of each dye as above. When both dyes were bound to the mutant, each dye was diluted to 4 mg mL^−1^ before addition to the bacteriophage to account for the increased conjugation capacity of the mutant expressing an additional dye-binding site per pVIII subunit.

Alexa Fluor 555 C_2_-maleimide or 647 C_2_-maleimide was dissolved in DMSO to 1.19 mg mL^−1^. To 1 mL of a 2 mg mL^−1^ solution of M13 bacteriophage in 50 mM potassium phosphate buffer, pH 8.0, 74 μL of a 20 mg mL^−1^ solution of *N*-succinimidyl *S*-acetylthioacetate (SATA) in DMSO was added (20-fold molar excess of SATA to M13 major coat protein pVIII). Following incubation for one hour at room temperature, 100 μL of 2.5 M hydroxylamine and 50 mM ethylenediaminetetraacetic acid (EDTA) in 50 mM potassium phosphate buffer pH 8.0 was added to quench the remaining SATA. The thiolated M13 was then separated from unbound reagents using a PD-10 de-salting column (GE Healthcare), eluting into 3.5 mL of 50 mM potassium phosphate, 150 mM NaCl, 5 mM EDTA, pH 7.0 (conjugation buffer), following manufacturer's instructions. The eluate was then diluted with 7 mL of conjugation buffer and to this, 210 μL of dye solution was added. The mixture was incubated for one hour in the dark at room temperature. To block free thiol groups, after the incubation with the dye, 80 μL of a 10 mg mL^−1^ solution of *N*-ethylmaleimide in deionised water was added to the mixture and allowed to incubate for 15 minutes in the dark at room temperature. To remove unbound reagents, polyethylene glycol (PEG) precipitation was performed. To the reaction mix, 5.5 mL of 25% PEG 6000 and 2.5 M NaCl in deionised water was added, before incubation at 4 °C in the dark for one hour to precipitate the M13-dye conjugate. The sample was then separated into 1.5 mL microcentrifuge tubes and centrifuged in a desktop centrifuge at full speed for 10 minutes. The supernatants were discarded and the pellets, containing the M13-dye conjugate, were suspended in a total volume of 2 mL of phosphate buffer.

### Bacteriophage-dye conjugate linear dichroism measurements

All bacteriophage-dye conjugate samples were diluted in 50 mM potassium phosphate buffer, pH 8.0. All LD measurements on the conjugates were made using a DIOP-0002 Ultra Low Volume Flow Linear Dichroism Accessory (non-thermostatted) (Dioptica Scientific, Rugby, UK), a 0.5 mm path length homemade quartz Couette cell rotating at 3000 rpm and a Jasco J-1500 Circular Dichroism Spectrometer with the parameters set to those detailed in the ESI.† All samples were baselined against 50 mM potassium phosphate buffer, pH 8.0 rotating at 3000 rpm.

### Bacteriophage mutagenesis

Mutations were introduced to the bacteriophage following standard site-directed mutagenesis procedures using: pVIII_F (3′ CTGTCTTTCGCTG**AG**GAG**AAA**GACGATCCCG 5′) and pVIII_R (3′ CGGGATCGTC**TTT**CTC**CT**CAGCGAAAGACAG 5′) mutagenic primers (altered nucleotides in bold);^33^ M13KE vector (New England Biolabs) as template; and pfu polymerase (Promega).

### Bacteriophage-dye conjugate fluorescence measurements

Neat bacteriophage variants labelled with Cy3 and Cy5 in 50 mM potassium phosphate buffer, pH 8.0 were loaded into a DIOP-0002 Ultra Low Volume Flow Linear Dichroism Accessory (non-thermostatted) (Dioptica Scientific, Rugby, UK) in a 0.5 mm path length homemade quartz Couette cell either stationary or rotating at 3000 rpm. Samples were illuminated using a Jasco J-1500 Circular Dichroism Spectrometer set at 540 nm with a 9 nm bandwidth. Fluorescence in the range of 250–700 nm was collected using an Ocean Optics HR2000 + CCD detector with a 1000 μm fibre optic cable attachment. The fibre optic cable was positioned to collect light at 100° from the incident light at the front face of the Couette cell. Spectra were recorded using Ocean Optics SpectraSuite software with an integration time of 6 s and a total accumulation of 24 scans. Spinning and non-spinning samples were baseline subtracted and are presented with a 4 nm 0th order Savitzky–Golay smoothing window. Error bars correspond to the standard deviation of measurements made in triplicate.

## Results and discussion

**Fig. 1:**
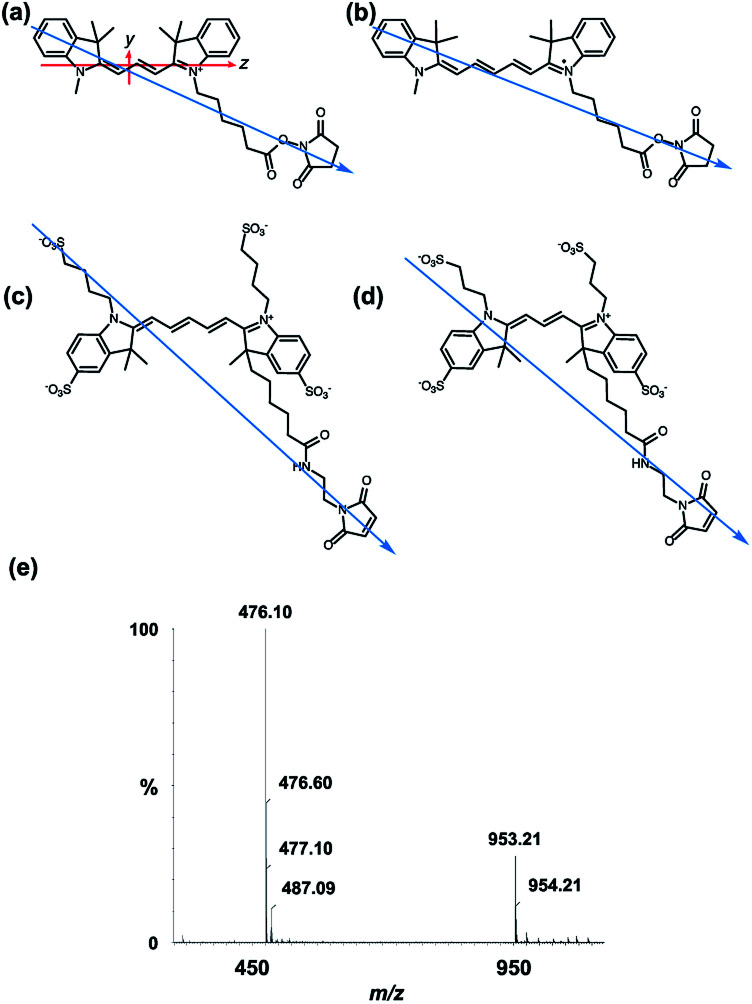
Dye structures. Molecular long axes denoted by blue arrow. (a) Cyanine3 NHS ester with long and short axes of the cyanine chromophore denoted with *z* and *y*, respectively. (b) Cyanine5 NHS ester. (c) Alexa Fluor 647 C_2_-maleimide. (d) Alexa Fluor 555 C_2_-maleimide. (e) Mass spectrum of Alexa Fluor 555 C_2_-maleimide.

All of the dyes used in this work are based on the same chromophore: Cyanines 3 and 5 (Cy3 and Cy5, respectively), and Alexa Fluors 555 C_2_-maleimide and 647 C_2_-maleimide (AF555 and AF647, respectively) are all based on the cyanine chromophore as illustrated in Fig. 1. AF555 is a proprietary dye whose structure is not in the literature. We therefore determined the mass of the dye (953.21 Da) and compared it to data on this dye family published in the patent database.^34^ This allowed us to assign the structure to the AF555 shown in Fig. 1e. Using the stretched-PE^ox^ film LD technique developed by Razmkhah *et al.*, we have characterised the transition dipole moment polarisations of the dyes in Fig. 1 and used this information to infer their orientations when bound to M13 bacteriophage using the microvolume Couette cell LD technique developed by Marrington *et al.*^31,35^ The dye orientation axis on the films is approximately the long axis illustrated in Fig. 1. As we orient the film stretcher so the long axis is horizontal, a positive signal means the transition polarisation is less than 54.7° from this axis. Since symmetry dictates that the dye chromophores have transitions approximately either along *z* or *y* (Fig. 1a). Bringing these two together, we can assign positive LD signals to *z* polarisation.

### Cyanine3 NHS ester

**Fig. 2:**
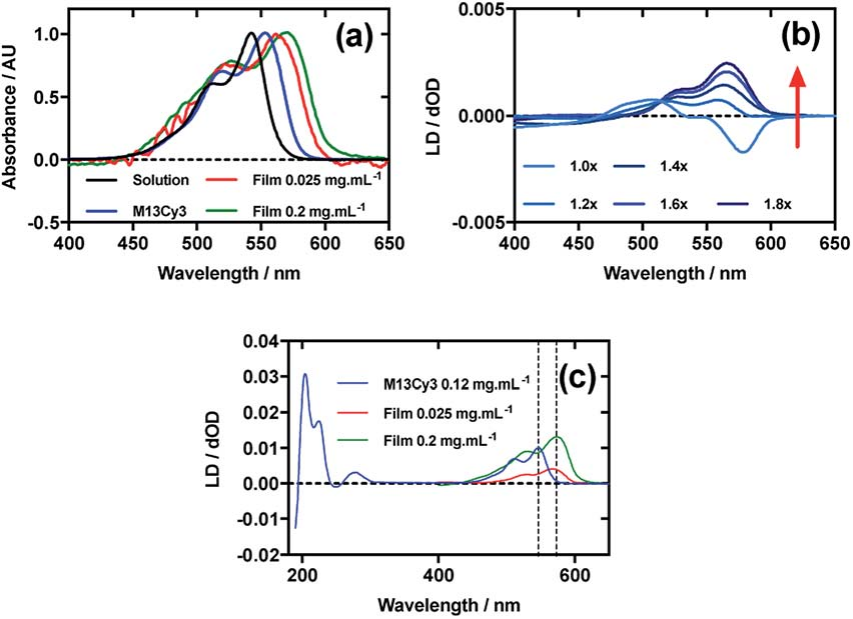
Linear dichroism spectra of Cyanine3 NHS ester and M13 bacteriophage Cy3 conjugate (M13Cy3). (a) Absorbance spectra of 2 μg mL^−1^ Cy3 in 50 mM potassium phosphate buffer, pH 8.0, dried onto PE^ox^ film from 0.2 mg mL^−1^ and 0.025 mg mL^−1^ solutions in 70 : 30 chloroform : methanol, and bound to M13 bacteriophage (M13Cy3) at 0.12 mg mL^−1^ in 50 mM potassium phosphate buffer, pH 8.0. (b) Stretched-film LD spectra of Cy3 at 0.025 mg mL^−1^, with increasing stretch factor of the film as indicated in the figure. Stretching was performed after deposition and drying of the dye. (c) Couette LD spectrum of M13Cy3 conjugate at concentration indicated in the figure in 50 mM potassium phosphate buffer, pH 8.0, and film LD spectrum of Cy3 from 0.025 mg mL^−1^ and 0.2 mg mL^−1^ solutions in 70 : 30 chloroform : methanol, with the film being stretched after deposition and drying of the dye sample.

Fig. 2a shows an overlay of Cy3 absorbance in solution, on film with a mainly monomer population (0.025 mg mL^−1^) (see LD discussion below), on film with a mixture of monomers and oligomeric structures (0.2 mg mL^−1^), and Cy3 on the bacteriophage (M13Cy3). The wavelength of maximum absorption (*λ*_A_max__) of Cy3 was bathochromically shifted relative to aqueous solution by 11 nm for M13Cy3, by 18 nm for the film monomer (deposited from 0.025 mg mL^−1^), and by 27 nm for the oligomeric structures on the film reflecting the increasing hydrophobicity of the Cy3 environments.

When Cy3 was deposited onto PE^ox^ after it had been stretched, the LD spectrum varied with stock solution concentration and contained bands that did not always correlate with the absorbance spectra: there were two negative bands at 505 nm and 547 nm and two positive bands at 445 nm and 585 nm in the LD spectra and just two bands at 527 nm and 570 nm in the absorbance spectra (data not shown) with different shaped spectra being observed for different amounts of sample deposited. The inconsistency of the wavelength and number of bands in the absorbance and LD spectra is consistent with the formation of higher order (at least dimeric) dye oligomers, albeit of unknown packing mode.^36^ When 30 μL of 0.025 mg mL^−1^ Cy3 was deposited onto un-stretched PE^ox^ and then stretched after the dye had dried (Fig. 2b) stepwise from unstretched (though with manufacturer's stretching) to 1.8×, the LD spectrum changed to produce an LD spectrum that more closely represented the shape of the absorbance spectrum on film (Fig. 2b), with two positive transitions at 522 nm and 561 nm. This change in LD with stretch factor suggests the disruption of dye oligomers to produce a population of more monomeric dye close to the orienting environment of the film surface. The LD of Cy3 deposited from 0.025 mg mL^−1^ at 1.8× stretch was closest to the absorbance in shape but still had a sloping LDr spectrum, which can be flattened by subtracting a small fraction (0.07) of the 0.1 mg mL^−1^ spectrum. Thus we conclude that the Cy3 is still somewhat oligomeric near the film even at full stretch from 0.025 mg mL^−1^. As the monomers will align with the long axis of the dye with the stretch direction, and the cyanine chromophore long axis is about 30° (much less than the magic angle of 54.7°) from the stretch direction we can conclude that the Cy3 band between 450 and 600 nm is polarised along the cyanine long axis.

Following the conjugation of Cy3 to M13 bacteriophage, the LD spectrum of the conjugate was measured, revealing the UV-region bands previously shown to be attributable to the bacteriophage as well as bands that the stretched-film experiments demonstrated were attributable to the Cy3 cyanine chromophore long axis.^37^ As Cy3 is too small to flow align by itself, its LD signal in the Couette setup (Fig. 2c) indicates successful conjugation. When the LD spectra of M13Cy3 at 0.015 mg mL^−1^ and 0.12 mg mL^−1^ are normalised at the wavelength of maximum LD (*λ*_LD_max__) in the region attributable to Cy3 (561 nm), the spectra match almost exactly (data not shown), indicating no formation of oligomeric dye structures or dye driven M13 assembly, and indicating that standard interpretation of the LD spectra may be applied even at the highest concentration used, which was not the case for the film LD experiment. The bacteriophage is known to align with its long axis in the direction of the applied flow, which in our setup was the same orientation as the stretch direction of the film. As the Cy3 bands are again positive, we conclude that Cy3 was bound with the long axis of its cyanine chromophore polarised more parallel than perpendicular to the long axis of M13 bacteriophage (Fig. 2c).

### Cyanine5 NHS ester

**Fig. 3:**
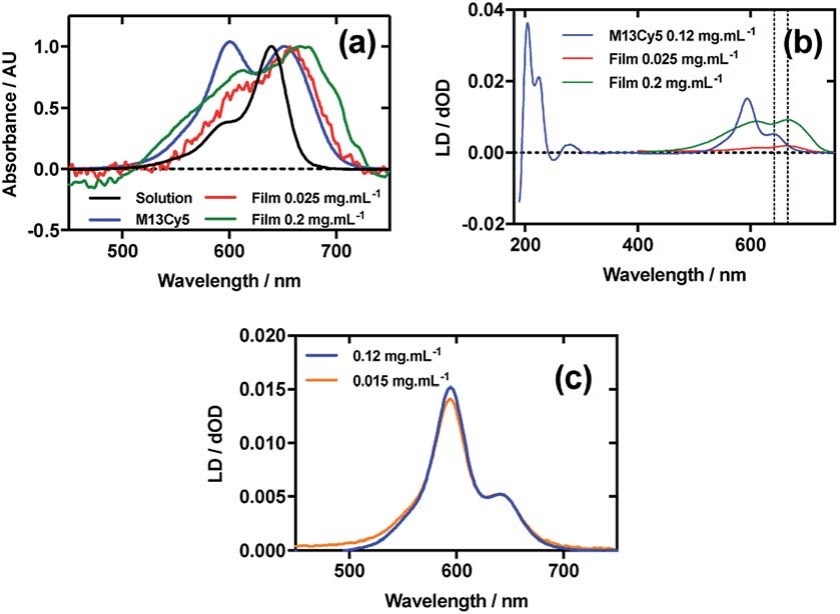
Linear dichroism spectra of Cyanine5 NHS ester and M13 bacteriophage Cy5 conjugate (M13Cy5). (a) Absorbance spectra of 2 μg mL^−1^ Cy5 in 50 mM potassium phosphate buffer, pH 8.0, dried onto PE^ox^ film from 0.2 mg mL^−1^ and 0.025 mg mL^−1^ solutions in 70 : 30 chloroform : methanol, and bound to M13 bacteriophage (M13Cy5) at 0.12 mg mL^−1^ in 50 mM potassium phosphate buffer, pH 8.0. (b) Couette LD spectrum of M13Cy5 at concentration indicated in the figure in 50 mM potassium phosphate buffer, pH 8.0, and film LD spectra of Cy5 from 0.2 mg mL^−1^ and 0.025 mg mL^−1^ solutions in 70 : 30 chloroform : methanol, with the film being stretched after deposition and drying of the dye sample. (c) LD spectra of M13Cy5 normalised at 651 nm, at concentrations indicated in the figure.

Fig. 3a shows an overlay of Cy5 absorbance in solution, on film with a mainly monomer population (0.025 mg mL^−1^), on film with a mixture of monomers and oligomeric structures (0.2 mg mL^−1^), and Cy5 on the bacteriophage (M13Cy5). The *λ*_A_max__ of Cy5 was bathochromically shifted relative to aqueous solution by 12 nm for M13Cy5, by 18 nm for the film monomer (deposited from 0.025 mg mL^−1^), and by 27 nm for the oligomeric structures on the film reflecting the increasing hydrophobicity of the Cy5 environments.

The dye bound to the bacteriophage appears to be more H-aggregated than the other samples measured, as indicated by the large peak at 600 nm, which is only present as a shoulder in the other samples. The larger relative magnitude of this band in the 0.2 mg mL^−1^ film spectrum relative to 0.025 mg mL^−1^ supports this interpretation. As free dye was removed from the solution after conjugation, the H-aggregates are assumed to form either between adjacent covalently bound Cy5 groups on the same virion or between Cy5 groups each bound to different virions. The 0.025 mg mL^−1^ stretched-film LD spectrum has two positive bands at 609 nm and 666 nm (Fig. 3b), approximately matching the film absorbance spectrum, which also has two bands at 609 nm and 657 nm (Fig. 3a and b). As monomeric Cy5 will align with the long axis of the dye with the stretch direction, and the cyanine chromophore long axis is about 30° (much less than the magic angle of 54.7°) from the stretch direction, we can conclude that the Cy5 band between 500 and 700 nm is polarised along the cyanine *z*-axis.

Following the conjugation of Cy5 to M13 bacteriophage, the LD spectrum of the conjugate was measured, as with Cy3, revealing the UV-region bands previously shown to be attributable to the bacteriophage as well as bands that the stretched-film experiments demonstrated were attributable to the Cy5 cyanine chromophore *z*-axis.^37^ As Cy5 is too small to flow align, its LD signal in the Couette setup indicates successful conjugation. In contrast to Cy3, when the LD spectra of M13Cy5 at 0.015 mg mL^−1^ and 0.12 mg mL^−1^ are normalised at the *λ*_A_max__ in the region attributable to Cy5 (651 nm), the spectra do not match perfectly, indicating the formation of oligomeric dye structures, and indicating that the standard interpretation of the LD spectra may not be applied at the highest concentration used, similar to the film LD experiment (Fig. 3c). The Cy5 bands are again positive, indicating that Cy5 was bound with the long axis of its cyanine chromophore polarised more parallel than perpendicular to the long axis of M13 bacteriophage (Fig. 3b) similar to the Cy3 case. However, its longer length linker has facilitated dye stacking. If the dye aggregates comprised dye groups bound to different virions, a loss of LD in the bacteriophage region compared to the other M13-dye conjugates would be expected due to the formation of multi-virion assemblies. However, this is not seen here so it is concluded that the dye aggregates are formed between dye groups on the same virion.

### Alexa Fluor 555 C_2_-maleimide

**Fig. 4:**
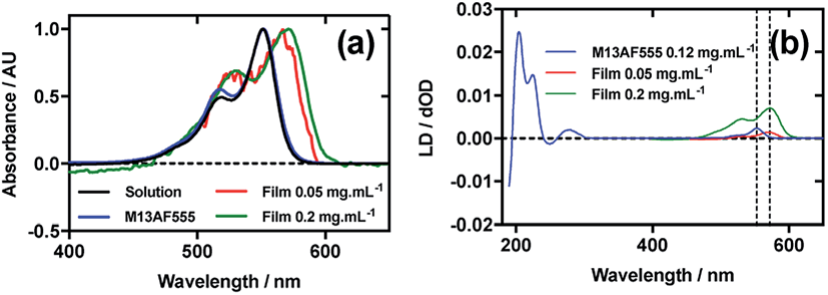
Linear dichroism spectra of Alexa Fluor 555 C_2_-maleimide and M13 bacteriophage AF555 conjugate (M13AF555). (a) Absorbance spectra of 2 μg mL^−1^ AF555 in 50 mM potassium phosphate buffer, pH 8.0, dried onto PE^ox^ film from 0.2 mg mL^−1^ and 0.05 mg mL^−1^ solutions in 70 : 30 chloroform : methanol, and bound to M13 bacteriophage (M13AF555) at 0.12 mg mL^−1^ in 50 mM potassium phosphate buffer, pH 8.0. (b) Couette LD spectrum of M13AF555 at concentration indicated in the figure in 50 mM potassium phosphate buffer, pH 8.0, and film LD spectra of AF555 from 0.2 mg mL^−1^ and 0.05 mg mL^−1^ solutions in 70 : 30 chloroform : methanol, with the film being stretched after deposition and drying of the dye sample.

Fig. 4a shows an overlay of AF555 absorbance in solution, on film at low loading concentration (0.05 mg mL^−1^), on film at high loading concentration (0.2 mg mL^−1^), and AF555 on the bacteriophage (M13AF555). The *λ*_A_max__ of AF555 on M13 bacteriophage was not shifted relative to aqueous solution, whereas it was bathochromically shifted by 20 nm for the low concentration film and 20 nm for the high concentration film reflecting the increasing hydrophobicity of these AF555 environments.

When a 0.4 mg mL^−1^ solution of AF555 was deposited onto un-stretched PE^ox^, which was then stretched after drying, the resulting LD spectrum did not correlate closely to the absorbance spectrum: the LD spectrum had two negative bands at 524 nm and 561 nm, and one positive band at 590 nm, whereas the absorbance spectrum had two bands at 530 nm and 571 nm (data not shown). The inconsistency in number and wavelength of the bands suggested aggregation of the dye. However, upon dilution of AF555 to concentrations of 0.2 mg mL^−1^ and lower, the resulting LD spectrum was similar in appearance to the film absorbance spectrum (Fig. 4b): the stretched-film LD spectrum had two positive bands at 531 nm and 572 nm, matching the film absorbance spectrum, which also had two bands at 531 nm and 572 nm (Fig. 4a and b). As monomeric AF555 will align the long axis of the dye with the stretch direction, and the cyanine chromophore long axis is about 30° (much less than the magic angle of 54.7°) from the stretch direction, we can conclude that the AF555 band between 450 and 600 nm is once again polarised along the cyanine long axis.

Following the conjugation of AF555 to M13 bacteriophage, the LD spectrum of the conjugate was measured, again revealing the UV-region bacteriophage-attributable bands as well as bands that the stretched-film experiments demonstrated were attributable to the AF555 cyanine chromophore long axis.^37^ As AF555 is too small to flow align, its LD signal in the Couette setup indicates successful conjugation. When the LD spectra of M13AF555 at 0.015 mg mL^−1^ and 0.12 mg mL^−1^ are normalised at the *λ*_LD_max__ in the region attributable to AF555 (553 nm), the spectra match almost exactly, indicating no formation of oligomeric dye structures, and indicating that standard interpretation of the LD spectra may be applied at the highest concentration used. The AF555 bands are again positive, indicating that AF555 was bound with the long axis of its cyanine chromophore polarised more parallel than perpendicular to the long axis of M13 bacteriophage (Fig. 4b).

### Alexa Fluor 647 C_2_-maleimide

**Fig. 5:**
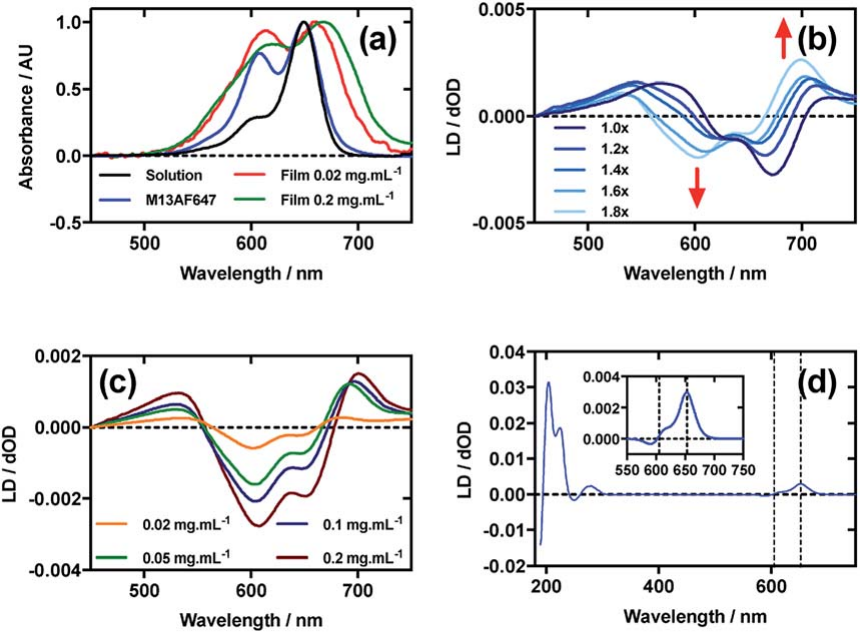
Linear dichroism spectra of Alexa Fluor 647 C_2_-maleimide and M13 bacteriophage AF647 conjugate (M13AF647). (a) Absorbance spectra of 2 μg mL^−1^ AF647 in 50 mM potassium phosphate buffer, pH 8.0, dried onto PE^ox^ film from 0.2 mg mL^−1^ and 0.02 mg mL^−1^ solutions in 70 : 30 chloroform : methanol, and bound to M13 bacteriophage (M13AF647) at 0.12 mg mL^−1^ in 50 mM potassium phosphate buffer, pH 8.0. (b) Stretched-film LD spectra of AF647 at 0.025 mg mL^−1^, with increasing stretch factor of the film as indicated in the figure. Stretching was performed after deposition and drying of the dye. (c) Stretched-film LD spectra of AF647 at concentrations indicated in the figure, stretching the film before dye deposition and drying. (d) Couette LD spectrum of M13AF647 at the concentration indicated in the figure in 50 mM potassium phosphate buffer, pH 8.0. Inset: bands attributable to AF647.

Fig. 5a shows an overlay of AF647 absorbance in solution, on film at low loading concentration (0.02 mg mL^−1^), on film at high loading concentration (0.2 mg mL^−1^), and AF647 on the bacteriophage (M13AF647). The *λ*_A_max__ of AF647 on M13 bacteriophage was not shifted relative to aqueous solution, whereas it was bathochromically shifted by 11 nm for the low concentration film and 19 nm for the high concentration film, reflecting the increasing hydrophobicity of the environment in which AF647 is located. When AF647 was deposited onto PE^ox^ after it had been stretched, at concentrations ranging from 0.02 to 0.2 mg mL^−1^, the resulting LD spectra did not correlate with the corresponding absorbance spectra (Fig. 5c). While the LD spectra had two positive transitions at 532 nm and 700 nm, and two negative transitions at 607 nm and 651 nm (Fig. 5c), the absorbance spectrum of film-dried AF647 had only two transitions at 619 nm and 668 nm (Fig. 5a). The discrepancy in the number and wavelength of transitions in the spectra suggests the formation of AF647 assemblies and the consequent exciton interactions, resulting in positive/negative LD couplets about the absorbance maxima. A similar effect was observed for Cy3 when deposited after the film was stretched, and for AF555 when deposited at 0.4 mg mL^−1^. For Cy3, this effect was removed by stretching the film after drying rather than before drying (Fig. 2b), and for AF555 this was removed by dilution, however these approaches were not effective for AF647 (Fig. 5b and c), thus it was not possible to determine the polarisation of the transition dipole moments of monomeric AF647 directly from the stretched-film LD data. However, as AF647, Cy3, Cy5 and AF555 share the cyanine chromophore, it can be inferred that the transition dipole moment of monomeric AF647 between 500 nm and 750 nm is polarised along the *z*-axis of the AF647 cyanine chromophore.

Following the conjugation of AF647 to M13 bacteriophage (M13AF647), the LD spectrum of the conjugate was measured, again revealing the UV-region bacteriophage-attributable bands as well as bands attributable to the AF647 cyanine chromophore *z*-axis.^37^ As AF647 is too small to flow align, its LD signal in the Couette setup indicates successful conjugation. When the LD spectra of M13AF647 at 0.015 mg mL^−1^ and 0.12 mg mL^−1^ are normalised at the *λ*_LD_max__ in the region attributable to AF647 (652 nm), the spectra match almost exactly, indicating no change in dye oligomerisation over the range of concentrations tested. However, as the absorbance spectra indicate the presence of dye oligomers (at least dimers), the standard interpretation of LD is not applicable. The AF647 bands at 615 and 653 nm are positive, indicating that they are polarised more parallel than perpendicular to the long axis of M13 bacteriophage (Fig. 5d). However, the band at 590 nm is negative indicating that this transition is polarised more perpendicular than parallel to the bacteriophage long axis.

**Fig. 6:**
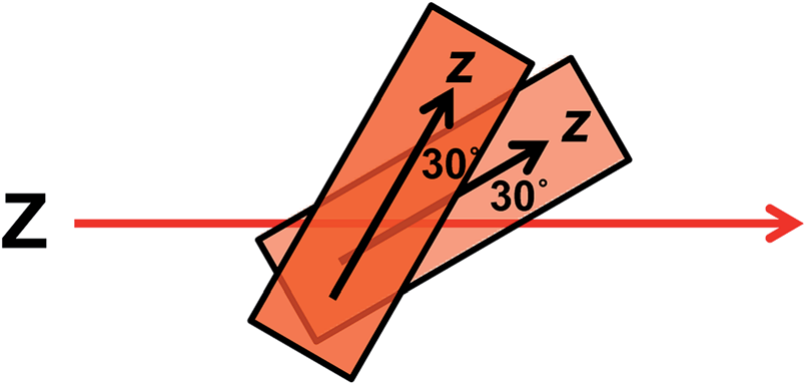
Proposed dimer geometry of Alexa Fluor 647. *Z* = orientation axis; *z* = cyanine chromophore long axis.

The wavelengths of the Couette LD signals suggest that at least some of the phage-bound dyes are managing to π–π stack. Given our inability to force AF647 on the films into monomeric form, it is clear that they have more affinity for each other than the dyes discussed above. So whether the π–π stacked dyes are all covalently bound or some covalent and some non-covalent we cannot determine. While the mode of packing of these dye assemblies is unknown, it is interesting to speculate about the geometry a dimer of AF647 might adopt to give rise to such patterns. One option is illustrated in Fig. 6, where dimers contain one AF647 molecule with its cyanine long axis (*z*) approximately 30° to the orientation axis (*Z*), and another AF647 molecule with its *z*-axis approximately 30° to the *z*-axis of the first.

Although only a small number of dyes were studied here, it is interesting to note any observed trends in the relationship between chemical structure and linear dichroism properties. We have noted in our laboratory in previous works that certain dyes either exhibit no LD or very weak LD when bound to M13 bacteriophage in the setup demonstrated here. These dyes include 4-chloro-7-nitrobenzofurazan (NBD-Cl), tetramethylrhodamine isothiocyanate (TRITC) and fluorescein isothiocyanate (FITC).^38^ These three dyes all have structures that are less elongated than the cyanines studied here, which could explain their weaker tendency to align on the bacteriophage. Interestingly however, no trend was observed here between the length of the dye molecules and their LDr values at wavelength of maximal absorbance, indicating that there are other factors that affect alignment, which are not apparent from the small number of dyes studied here. It was observed, however, that all dyes tested (except AF647 as monomerisation could not be achieved) exhibited stronger LDr when aligned using film than bacteriophage, with values of the order of 0.15–0.23 and 0.03–0.09, respectively. This discrepancy could reflect the fact that the dyes bound to the bacteriophage are in solution and thus are less rigidly aligned than the dyes dried onto film.

### LD to inform FRET optimisation

It is well established that Förster resonance energy transfer (FRET) efficiency is dependent on the alignment of the TDMs of the donor and acceptor groups thus it was reasoned that optimisation of dye arrays used to create biomimetic light-harvesting antennae would require alteration of the surface geometry of the scaffold used as well as alteration of the separation of the donors and acceptors.^39^ To monitor the relative alignment of the donor/acceptor TDMs, LDr could be measured. Theoretically, if the two dyes' TDMs were polarised to the same axis, their LDr values would be equal so the ratio of the two would be 1, and their FRET efficiency would be maximised at the given distance. This would be advantageous to monitor during optimisations, as when only FRET is measured, effects on FRET efficiency due to donor–acceptor misalignment, rather than separation, cannot be measured directly.

To demonstrate this, we used an M13 mutant with lysine at position 3 in pVIII coat protein, and therefore an additional alternative conjugation site for dyes to bind.^33^ It was therefore expected that the mutant should bind dyes in two different orientations (and wild type only one, the amino-terminus of the bacteriophage major coat protein), and therefore should have lower correlation in LDr of the two dyes bound and therefore lower FRET efficiency than wild type even when the donor and acceptor groups are bound to the same extent to the two bacteriophage variants.

As LD is measured under shear flow to align the bacteriophage, FRET was also measured under shear flow to ensure that the alignment of the dyes relative to the bacteriophage and to each other was the same for both measurements. FRET was also measured in non-aligning conditions *i.e.* with no shear flow, to ascertain whether there was an alteration in the dye alignment upon application of shear flow, which would be indicated by any alteration in FRET.

**Fig. 7:**
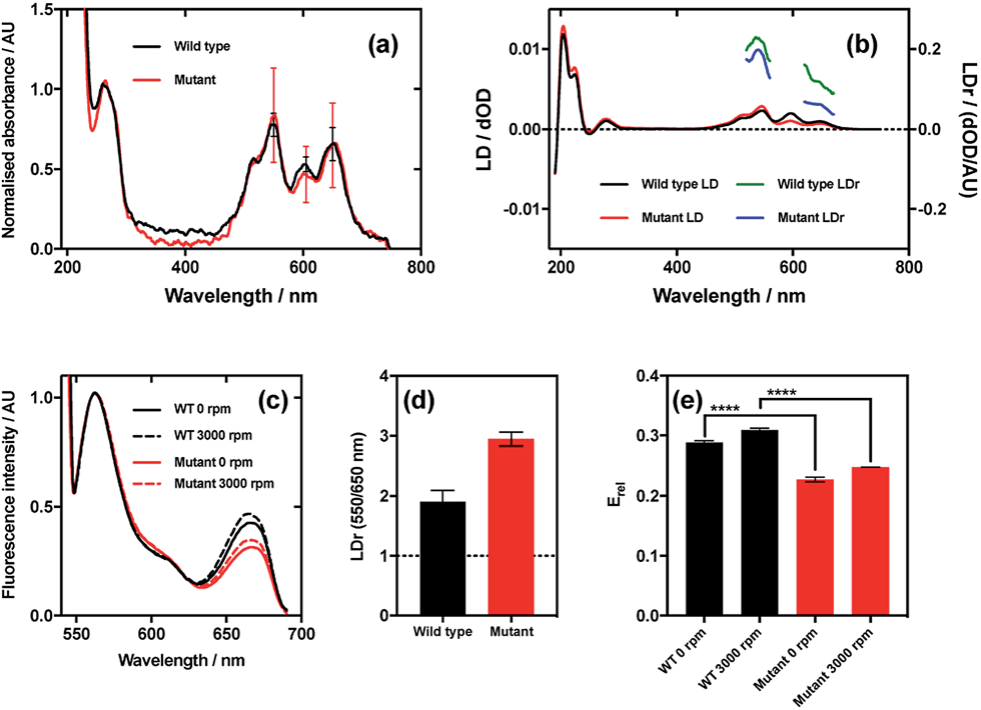
Relationship between dye relative alignment and FRET efficiency. (a) Absorbance spectra of wild type and mutant M13 bacteriophages labelled with Cy3 and Cy5. (b) LD and reduced LD (LDr) of wild type and mutant bacteriophage conjugates. (c) FRET emission spectra of wild type and mutant bacteriophage conjugates stationary (0 rpm) and under shear flow (3000 rpm). Band at 550 nm due to Cy3 (donor) emission, and band at 650 nm due to Cy5 (acceptor) emission. (d) Ratio of LDr at regions attributable to Cy3 and Cy5 (LDr 550/650 nm) of wild type and mutant bacteriophage conjugates. Line at ideal value of 1. (e) Ratiometric FRET efficiencies of dyes bound to wild type and mutant bacteriophages stationary and under shear flow.

The results of this experiment indicate that even though the wild type and mutant bacteriophages bound Cy3 and Cy5 (donor and acceptor, respectively) to the same extent (Fig. 7a), the LD signals of the dyes were clearly different, resulting in different LDr in the regions attributable to Cy3 and Cy5 (Fig. 7b). When the ratio of LDr at 550 nm (Cy3) and 650 nm (Cy5) (LDr^550/650^) is calculated, the result for wild type is 1.9 and the result for the mutant is 2.9 (Fig. 7d). The ideal situation, *i.e.* when the TDMs of the donor and acceptor are aligned about the same axis, would result in an LDr^550/650^ of 1, thus in this case the dyes bound to the wild type are more similarly aligned than those on the mutant. It would thus be expected that the FRET efficiency of the dyes on the wild type would be greater than that of the dyes on the mutant. When the FRET was measured in aligning conditions (Fig. 7c, 3000 rpm) and the FRET efficiency was calculated, this was indeed the case. The fluorescence spectra of the two variant conjugates revealed greater fluorescence intensity due to the acceptor (Cy5; band at 660 nm) in the wild type than the mutant (Fig. 7c), indicating higher FRET efficiency. To quantify the difference, relative FRET efficiency (*E*_rel_ = *I*_650_/(*I*_550_ + *I*_650_), where *I* is the fluorescence intensity at the wavelength indicated by the subscript number) was calculated (Fig. 7e, 3000 rpm), revealing that the *E*_rel_ of the dyes bound to wild type was significantly higher (*p* < 0.0001) than that of those on the mutant, with values of 0.31 ± 0.02 and 0.25 ± 0.002, respectively, a 24.9% loss of FRET efficiency attributable, by measuring LD, to donor–acceptor misalignment.

When FRET was measured in non-aligning conditions (Fig. 7c and e, 0 rpm), the band attributable to the acceptor dye, Cy5 (660 nm), was less intense than under shear flow. This was the case for both mutant and wild type bacteriophages. The absolute difference in *E*_rel_ between the wild type and the mutant in non-orienting conditions was the same as under shear flow, with both losing 0.06, indicating that the perturbation in FRET caused by the mutation is preserved in both conditions. The fact that there was a change in FRET upon application of shear flow indicates that the dyes can rotate relative to the bacteriophage scaffold. The fact that the FRET efficiency increased upon application of shear flow indicates that the dyes became better aligned relative to each other under shear flow. Combining the fact that the FRET efficiency increased upon application of shear flow and the observation that the dyes align with their long axes more parallel than perpendicular to the bacteriophage long axis (LD experiments under shear flow) suggests that the dyes become more highly aligned to the bacteriophage long axis upon application of shear flow. The behaviour of the dyes on the bacteriophage is thus analogous to that of a flag on a moving vehicle.

## Conclusions

We have employed stretched-film LD spectroscopy on oxidised polyethylene to assign the TDM polarisations of a range of cyanine dyes. Following this we have been able to determine the alignment of the dyes when they are bound to M13 bacteriophage, a model linear scaffold for the generation of biomimetic light-harvesting antennae relying on FRET between donor and acceptor dyes. Furthermore, we have shown how LD spectroscopy may be used to distinguish between lack of FRET efficiency on the antennae due to dye separation and dye misalignment. This will enable rational design and optimisation of biomimetic light-harvesting antennae based upon linear scaffolds.

## Conflicts of interest

There are no conflicts to declare.

## Supplementary Material

RA-008-C8RA05475D-s001
