# Supplementary material for: Linear dichroism of visible-region chromophores using M13 bacteriophage as an alignment scaffold
Source: RSC Adv. 2018 Aug 20;8(52):29535–43. doi: 10.1039/c8ra05475d (PMC6333254; doi:10.1039/c8ra05475d)
Supplement: RA-008-C8RA05475D-s001 [file RA-008-C8RA05475D-s001.pdf]

Electronic Supplementary Material (ESI) for RSC Advances.
This journal is © The Royal Society of Chemistry 2018

Supplementary Information

Linear dichroism of visible-region chromophores using M13 bacteriophage as an alignment scaffold

Matthew Tridgett, Charles Moore-Kelly, Jean-Louis H. A. Duprey, Lorea Orueta Iturbe, Chi W. Tsang, Haydn A. Little, Sandeep K. Sandhu, Matthew R. Hicks, Timothy R. Dafforn and Alison Rodger

S1 Linear dichroism parameters

Table S1 Linear dichroism parameters

Parameter	Cy3	Cy5	Alexa Fluor 555	Alexa Fluor 647	M13Cy3	M13Cy5	M13AF555	M13AF647
Wavelength start / nm	650	750	650	750	650	750	650	750
Wavelength end / nm	190	190	190	190	190	190	190	190
Data interval / nm	1	1	1	1	1	1	1	1
Data pitch / nm	1	1	1	1	1	1	1	1
Digital integration time / s	1	1	1	1	1	1	1	1
Bandwidth / nm	2	2	2	2	2	2	2	2
Scanning speed / $nm.min^{-1}$	200	200	200	200	200	200	200	200
Accumulations	2	2	2	2	2	2	2	2
